# Prevalence and significance of incidental findings on 68 Ga-DOTA-conjugated somatostatin receptor-targeting peptide PET/CT: a systematic review of the literature

**DOI:** 10.1186/s40644-022-00484-0

**Published:** 2022-09-03

**Authors:** Morten Bentestuen, Farid Gossili, Charlotte Elberling Almasi, Helle Damgaard Zacho

**Affiliations:** 1 Department of Nuclear Medicine and Clinical Cancer Research Center, Aalborg University Hospital, Hobrovej 18-22, 9000 Aalborg, Denmark; 2 Department of Clinical Medicine, Aalborg University, Aalborg, Denmark

**Keywords:** SSTR PET/CT, Ga-68 DOTA PET/CT, Incidentalomas, Incidental findings, Risk of malignancy, False positives

## Abstract

**Aim:**

We aimed to evaluate the prevalence of incidental ^68^ Ga-DOTA-conjugated somatostatin receptor-targeting peptide PET/CT (SSTR PET/CT) findings, their clinical significance in the need for follow-up, and their risk of malignancy.

**Materials and methods:**

Studies reporting incidental SSTR PET/CT findings were systematically searched in PubMed, Cochrane, Embase and Web of Science literature published prior to 1^st^ of May 2020. Studies were filtered by two independent readers for eligibility based on title and abstract, and subsequently on full text. The main exclusion criteria were: 1) pathological findings that matched scan indication, 2) known organ specific disease and/or incidental findings confirmed on other scan modality prior to SSTR PET/CT, 3) lack of diagnosis and/or follow up, and 4) results published in proceedings or conference abstracts.

**Results:**

Twenty-one studies, comprising a total of 2906 subjects, were eligible for the analysis. Studies included were retrospective cohort studies on incidental SSTR PET/CT findings in a specific organ (*n* = 2888, 7/21) or case reports (*n* = 18, 14/21). A total of 133 subjects had incidental SSTR PET/CT findings. Incidental findings were predominantly seen in the thyroid gland (*n* = 65), spine (*n* = 30), brain (*n* = 26) and breast (*n* = 6). Seventeen of 133 (13%) incidental findings were malignant on final diagnosis. Incidental breast findings were associated with the highest risk of malignancy (67%). In the thyroid, incidental SSTR uptake was caused by malignancy in 8%, all presenting as focal uptake. The lowest risk was seen in the spine with a malignancy rate of 3% in patients with incidental SSTR uptake and benign cases were interpreted as vertebral hemangiomas on CT. Incidental SSTR PET/CT findings in other locations were of malignant etiology in two out of six cases (33%) and should be evaluated individually.

**Conclusion:**

The most incidental SSTR PET/CT findings were found in the thyroid gland, spine, and brain. The risk of malignancy was greatest in incidental SSTR PET/CT findings in the breast, cranially, and thyroid gland. The results of the present study can prove useful in the interpretation of atypical findings on SSTR PET/CT and in the counseling of clinicians.

**Supplementary Information:**

The online version contains supplementary material available at 10.1186/s40644-022-00484-0.

## Introduction/background

Somatostatin receptors (SSTR) subtype 1–5 belong to the G-protein coupled receptor superfamily and normally have a wide variety of physiological functions in the body, from the regulation of various hormones and neuropeptides, gastric emptying, and intestinal blood flow [1]. Neuroendocrine tumors (NET), a relatively heterogeneous group of tumors mainly arising in the gastro-entero-pancreatic tract (75%) and lungs (25%), are known to express SSTR, most abundantly SSTR2 that is expressed in 70–90% of all NETs [2]. This phenomenon has been utilized in diagnostic imaging with positron emission tomography (PET) and also in SSTR-targeting radionuclide treatment [3]. SSTR-targeting peptides linked with the 1,4,7,10-tetraazacyclodecane-1,4,7,10-tetraacetic acid (DOTA) with a chelated positron emitting radioactive isotope enables PET imaging of NET with high sensitivity and specificity [4, 5]. Gallium-68 is the most commonly used isotope since it has favorable properties for diagnostic imaging and can be produced onsite from a generator without a cyclotron. Different tracers have been developed by modifications of the SSTR binding Tyr3-Octreotide with different affinities towards the SSTR subtypes [2, 6]. Although DOTA-tracers show a reliable specificity in oncologic PET-imaging on known or suspected NETs, physiological uptake of DOTA-tracers is considerable in the liver, adrenal glands, urinary tract, pancreas, spleen, and pituitary gland, and modest in the gastrointestinal tract, thyroid- and salivary glands [7–10].

In the last decade SSTR PET/CT scans are increasingly performed and therefore, encountered atypical or incidental findings (or incidentalomas) are expected to increase in the future [2]. The understanding of SSTR-PET/CT incidentalomas is important for the evaluation and interpretation of SSTR-PET/CT. Additionally, clinicians need to know how to deal with unexpected findings to avoid unintended consequences. However, literature on the incidence as well as outcomes of SSTR PET/CT incidentalomas are scarce and has predominantly focused on incidental SSTR uptake in specific organs such as the thyroid gland [11, 12].

We aimed at evaluating the overall prevalence and risk of malignancy in incidental SSTR PET findings published in the literature.

## Materials and methods

### Study design

We conducted a systematic review of observational studies describing the prevalence and outcomes of incidental findings reported on ^68^ Ga-SSTR PET/CT or PET/MRI. The systematic review was performed in accordance with the Preferred Reporting Items for Systematic Reviews and Meta-Analysis (PRISMA) guideline [13].

### Search strategy and eligibility criteria

A comprehensive literature search of PubMed, Embase, Web of Science, and The Cochrane Library was conducted. The search period was from the start of each database until 1^st^ of May 2020. Databases were searched by using headings under the terms: incidental finding, unexpected finding AND DOTA-ligand PET/CT or PET/MRI (search profiles are presented in supplementary material). To expand our search, references of the retrieved articles were also screened for additional studies. All data were managed using the reference managing tool EndNote Web 3.3.

According to the report characteristics such as year of publication, language and country of first author, and according to the study characteristics such as PICOS concept (patient, intervention, comparator, outcome, study type), the following inclusion criteria were used: 1. Incidental ^68^ Ga-DOTA-conjugated Somatostatin Receptor-Targeting (SSTR) Peptide PET/CT finding, 2. Original articles published in peer reviewed journals, 3. No restrictions with respect to language, geographical limits or date, and 4. No restrictions regarding study design, single cases (including illustrative examples in reviews and editorials) were included. A SSTR PET incidentaloma was defined as findings unknown prior to SSTR PET *or* conditions with unknown potential uptake of SSTR-targeting tracers.

The following predefined exclusion criteria were used: 1. Pathological findings that matched scan indication, 2) Known organ specific disease and/or incidental findings confirmed on other scan modality prior to SSTR PET, 3. Studies not published in peer review (i.e., conferences, annual meetings etc.), and 4. Articles and/or cases with insufficient information and/or lack of confirmed diagnosis/pathology.

The inclusion and exclusion criteria were utilized on both a study and case level.

Two physicians (MB and FG) independently reviewed the titles and abstracts of the retrieved articles. The same two physicians then independently reviewed the full-text version of the remaining articles to determine their eligibility for inclusion. Disagreements were resolved by a third author (CA or HZ).

### Data extraction

The following data were extracted from the included studies: author, publication year, impact factor of journal, country, study design, imaging indication, choice of tracer, organ of incidental findings, number of patients included in respective study, number of patients with sufficient follow-up, and the confirmed diagnosis. This data was extracted by one reviewer (MB) and controlled by a second reviewer (FG).

In cases where it was unclear whether a reported finding was incidental, or there was a lack of confirmational diagnostics, the reported finding was excluded from the analysis. These unclear cases were discussed between all of the authors (MB, FG, CA and HZ).

### Synthesis

Incidental findings were grouped based on the anatomical location of the respective incidental findings. Incidental findings were then grouped as malignant or benign based on final confirmed diagnosis and/or pathology.

### Statistics

Statistics in this review are descriptive. Due to many included case-studies and small sample size studies, no analysis of quality of evidence was conducted. Regarding reported incidental findings, no statistical meta-analysis was conducted due to few cohort studies and due to heterogeneity in study design.

## Results

### Included studies and study demographics

The systematic literature search identified 395 individual publications from four databases, which was reduced to 259 papers after removal of duplicates. A total of 195 papers were rejected based on title and abstract, and 64 reports were retrieved for full-text reading.

Forty-three studies were excluded after a full text readthrough.

**Fig. 1:**
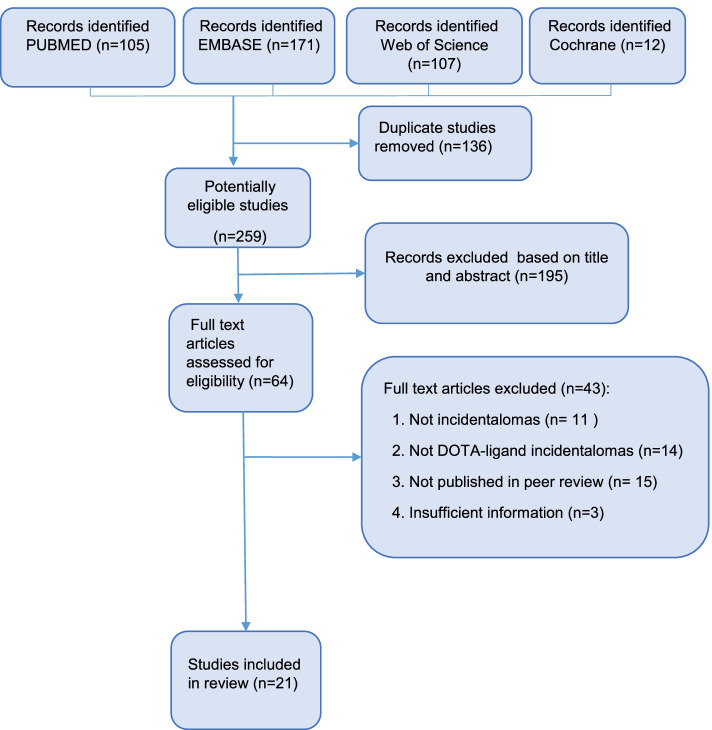
Flow chart search and screening

Finally, 21 papers were included in the present systematic review. The search and inclusion process are summarized in Fig. 1. All studies were published in English. The majority of the studies were conducted in Europe and North America. Studies included were retrospective cohort studies on incidental SSTR PET/CT findings in a specific organ (*n* = 2888, 7/21) or case reports (*n* = 18, 14/21). A total of 2906 study subjects were included. Most included study subjects (*n* = 2888, 99%) were from cohort studies, the remaining subjects were from case reports (*n* = 18, 1%). Among the study subjects, incidental findings were reported in 187 patients (6%). Finally, a total of 133 patients had sufficient diagnostic follow-up and were included in the final analysis: 16 patients were from case reports and 117 patients were from cohort studies (Tables 1 and 2).

**Table 1:** Results study characteristics

Variable	Result
Year of publication, median (range)	2016 (2008–2020)
Number of authors, median (range)	5 (2–10)
Geographical origin, n (%)	
Europe	10 (48%)
Asia	3 (14%)
North America	4 (19%)
South America	1 (5%)
Australia	3 (14%)
Africa	0
Nuclear medicine affiliation, n (%)	
Yes	20(95%)
No	1(5%)
Study design, n (%)	
Cohort studies	7 (33%)
Number of patients, median (range)	412 (33–1150)
Case reports	14 (67%)
^68^ Ga-Tracer of choice, n (%)	
DOTATATE	15 (71%)
DOTATOC	4 (19%)
DOTANOC	2 (10%)
Impact factor, n (%)	
Journals without impact factor	3 (14%)
Journals with impact factor	18 (86%)
Impact factor, median (range)	4,241 (1.085–6.622)

**Table 2:** Overview included studies and incidental findings included and synthesized in our review

Author	Year	Country	Tracer	Study design	Number of findings included and synthesized in review	Findings included and synthesized in our review
Elgeti	2008	Germany	Ga-68-DOTATOC	Cohort, retro	2	2 Invasive ductal breast cancer
Law	2013	Australia	Ga-68-DOTATATE	Case report	1	1 Meningioma
Kuyumcu	2013	Turkey	Ga-68-DOTATATE	Cohort, retrospective	3	1 Breast fibroadenoma 1 Meningioma 1 Fibrous Dysplasia
Skoura	2015	UK	Ga-68-DOTATATE	Case report	1	1 Vertebral hemangioma
Kunikowska	2015	Poland	Ga-68-DOTATATE	Cohort, retrospective	46^a^	14 Hashimotos 14 Goiter 10 Physiological / nodular thyroid gland 8 Hashimotos + goiter 1 Papillary thyroid carcinoma^a^
Chan	2016	Australia	Ga-68-DOTATATE	Case report	1	1 Medulloblastoma cerebrum
Cleary	2016	UK	Ga-68-DOTATATE	Cohort, retrospective	10	4 Meningioma 6 Physiological uptake
Lasocki	2016	Australia	Ga-68-DOTATATE	Case report	1	1 Cerebellar hemangioblastoma
Has Simsek	2016	Turkey	Ga-68-DOTATATE	Case report	1	1 Solitary bone plasmacytoma
Nockel	2016	USA	Ga-68-DOTATATE	Cohort, retrospective	16	8 Thyroid adenomas 4 Goiter 3 Papillary thyroid carcinoma 1 Physiological / nodular thyroid gland
Yamaga	2017	Brazil	Ga-68-DOTATATE	Case report(s)	3	1 Non-Hodgkins lymphoma 1 Breast carcinoma 1 Thyroid adenoma
Vadrucci	2017	Italy	Ga-68-DOTATOC	Case report	1	1 Retiform hemangioendothelioma
Mahajan	2017	USA	Ga-68-DOTATATE	Case report	1	1 Medullary thyroid carcinoma
Sampaio	2018	Portugal	Ga-68-DOTANOC	Case report	1	1 Breast cancer
Arora	2018	India	Ga-68-DOTANOC	Case report	1	1 Parathyroid adenoma
Laurens	2018	Netherlands	Ga-68-DOTATOC	Case report	1	1 Pleomorphic parotid adenoma
Ishiyama	2018	USA	Ga-68-DOTATATE	Case report	1	1 Elastofibroma dorsi
Gauthe	2018	France	Ga-68-DOTATOC	Cohort, retrospective	28	28 Vertebral hemangiomas
Parghane	2019	India	Ga-68-DOTATATE	Cohort, retrospective	12	6 Meningiomas 4 Skull base paragangliomas (metastatic) 2 Pituitary adenomas
Pandika	2019	USA	Ga-68-DOTATATE	Case report	1	1 Gynecomastia
Liu	2020	China	Ga-68-DOTATATE	Case report	1	1 Uterine leiomyoma

^**a**^ Note: one the thyroid incidental SSTR PET/CT findings are reported twice since one lesion was initially reported as benign and later found to be malignant

### Imaging characteristics

^68^ Ga-DOTATATE was the most common tracer (15 out of 21 studies. 71%) followed by ^68^ Ga-DOTATOC (*n* = 4, 19%) and ^68^ Ga-DOTANOC (*n* = 2, 10%). All studies were conducted using a PET/CT-system.

In most studies, the scan indication was known or suspected neuroendocrine tumor (NET) (*n* = 15, 71%), comprising a total of 2896 patients (99.7%). In the final patient selection, 125 out of 133 patients with incidental SSTR PET findings (94%) were scanned on this indication. In the remaining 8 cases, the scan indication was not presented in the paper.

### Results: incidental findings

On a case level, most incidental SSTR PET findings were seen in the thyroid gland (*n* = 65, 49% of all incidental findings), followed by spine (*n* = 30, 23%), cranial findings (*n* = 26, 20%), breast (*n* = 6, 4%) and miscellaneous locations (*n* = 6, 4%) (Table 2).

### Thyroid

Four studies (19%) assessed or contained cases of incidental thyroid SSTR PET/CT uptake [11, 12, 14, 15]. Incidental uptake in the thyroid gland was the most prevalent of all included incidental SSTR PET/CT findings. A total of 65 patients had incidental uptake in the thyroid gland: 25 patients had focal uptake and 40 patients had diffusely elevated uptake (Table 3). In the retrospective cohort studies by Nockel et al. and Kunikowska et al. the incidence of thyroid incidental findings on SSTR PET was estimated to be 11% (26/237) and 4% (46/1150), respectively [11, 12].

**Table 3:** Pooled prevalence and diagnosis of thyroid SSTR PET incidentalomas^a^

Thyroid Gland		
**Total: 65**	**Focal: 25**	**Diffuse: 40**
Malignant	5	0
Thyroid adenomas	7	2
Hashimotos	1	13
Goiter	5	13
Hashimotos + Goiter	6	2
Physiological / nodular thyroid	1	10

One the thyroid incidental SSTR PET findings are reported twice since one lesion was initially reported as benign and later found to be malignant

^a^ Some of the patients had known thyroid disease

Incidental focal SSTR-uptake was proven to be due to malignancy in five of 25 cases with focal uptake (20%): Four cases of papillary carcinomas and 1 case of medullary carcinomas were revealed [11, 12, 14]. The remaining patients with focal incidental SSTR-uptake were consistent with adenoma (*n* = 7, 28%), multinodular goiter (*n* = 5, 20%), Hashimotos and coexisting multinodular goiter (*n* = 6, 24%), Hashimotos (*n* = 1, 4%), and finally physiological, nodular thyroid gland (*n* = 1, 4%) [11, 12, 15]. No malignancy was found with supplementary diagnostics in patients with diffuse elevated uptake (Table 3) [11, 12, 15].

### Cranial

A total of 26 cranial SSTR PET incidentalomas (20%) were reported. Five cases (19%) were proven to be malignant: 4 cases of skull base paragangliomas (15%) and 1 case of cerebellar medulloblastoma (4%) [16, 17].

In benign cases, meningiomas were the most frequent finding (*n* = 12, 46%) [8, 17–19]. In the study by Cleary et al. (2016), six incidental SSTR PET findings were without clear anatomical structures on supplementary diagnostic imaging and were hypothesized to be due to physiological uptake in veins (*n* = 3) and sinuses (*n* = 2); however, the last incidental finding was of unknown etiology in the right frontal lobe (SUVmax 5.82) without correlating lesion on non-contrast MRI (Table 4) [20].

**Table 4:** Pooled prevalence and diagnosis of cranial SSTR PET incidentalomas

Cranial	Total 26	Type of malignancy
Malignant	5	4 Paraganglioma (metastastatic)^a^ 1 Cerebellar medulloblastoma
Meningioma	12	
Pituitary adenoma	2	
Physiological	5	
Unknown etiology	1^b^	
Cerebellar Hemangioblastoma	1	

^a^ Described as malignant metastases by the author of the article

^b^ Focal uptake in right frontal lobe without correlating lesion on non-contrast MRI

### Breast

Our search revealed four studies reporting a total of six cases of incidental SSTR PET uptake in the breast with sufficient follow-up. On further diagnostics, four out of six incidentalomas (67%) were of malignant origin (two invasive ductal carcinomas and two patients with breast cancer not further specified), and in one of these cases, metastases to axillary lymph nodes were visible on SSTR PET/CT [15, 21, 22]. Benign incidental findings were asymmetric idiopathic gynecomastia (*n* = 1) and breast fibroadenoma (*n* = 1) [8, 23].

### Spine

Regarding incidental spine SSTR PET findings, our study revealed 1 cohort study and 2 case reports.

In the case study by Has Simsek et al., the lesion of the spine was proven to be a malignant solitary bone plasmacytoma on further diagnostics [24]. In the single center cohort studies by Gauthe et al. (2018), a total of 28 patients with incidental spine SSTR PET findings were consistent with vertebral hemangiomas on diagnostic imaging [25]. The case report by Skoura et al. had similar results with a spine-lesion proven to be vertebral hemangioma [26].

### Miscellaneous

Studies reporting incidental SSTR PET uptake in other locations were case studies (*n* = 7). Two cases proved to be malignant (29%), including retiform hemangioendothelioma in soft tissue and a non-Hodgkins lymphoma in a lymph node [15, 27]. The remaining five benign cases revealed a pleomorphic adenoma in the parotid gland, parathyroid adenoma in close relation to the thyroid gland, elastofibroma dorsi in soft tissue, a uterine leiomyoma, and fibrous dysplasia in the femoral bone [8, 28–31].

## Discussion

This is – to the best of the authors’ knowledge—the first review that aimed to assess incidental SSTR PET/CT findings. Based on the cohort studies, the overall incidence of incidental SSTR PET findings was approximately 4%.

The thyroid gland was the most frequently reported location of incidental SSTR uptake with an estimated incidence of between 4 and 11% in patients with known or suspected NET. In almost two thirds of the cases with unexpected thyroid uptake, the SSTR uptake was diffusely elevated, and no malignant cause was found on further investigations. In contrast, focal uptake in the thyroid carried a 20% risk of malignancy. These results imply that all incidental focal thyroid findings should be further investigated. Incidental thyroid uptake is also detected in approximately 2% of all FDG PET/CTs and 4% of all PSMA PET/CTs [32, 33]. Similarly, focal thyroid uptake on FDG PET/CT is associated with a high risk of malignancy (35%) whereas it is approximately 4% in diffuse uptake. For PSMA PET/CT, the risk of malignancy for focal uptake is 15% and no cases of malignancy in diffuse uptake have been reported [32–34].

Many of the included patients with incidental thyroid SSTR uptake had known disease of the thyroid gland prior to SSTR PET/CT. This may cause bias in the population, however, we only included cases included in the review which exhibited much higher SSTR uptake than normal physiologic thyroid uptake which had been deemed as abnormal by the interpreting nuclear medicine physician, for example Nockel et al. defined increased thyroid SSTR uptake as SUVmax above liver or salivary gland [12]. If such information was not available the cases were excluded. Likewise, cases of known thyroid disease were excluded if they were reported to have been confirmed with another scan modality prior to SSTR PET and/or known to be malignant prior to PET.

It is worth mentioning that a few studies were not included in the final synthesis due to their results not being published in peer reviewed journals or due to insufficient information. These studies reported additional incidental thyroid findings e.g. Sobral-Violante et al., found seven cancers among 16 incidental thyroid findings [35].

Following incidental thyroid uptake, SSTR uptake in the spine and cranial region were the most common localizations of incidental findings. Not surprisingly, meningiomas were the most frequent intracranial finding. In our study, we defined meningiomas as benign findings since in most cases they are low grade type 1 neoplasms, as defined by the World Health Organisation, and only very few are atypical, higher grade meningiomas [36]. It should therefore be stressed that incidental findings resembling meningiomas should always be followed up in accordance with standard regimens. Similarly, pituitary adenomas were defined as benign with elevated SSTR uptake on PET, however no further details regarding the incidental pituitary findings were reported. In other studies, pituitary adenomas and non-functioning pituitary adenomas have lower uptake on SSTR PET/CT indicating that elevated SSTR uptake is common in normal pituitary tissue [37, 38]. Of interest, SSTR PET/CT has also been valuable in localizing ectopic Cushing syndrome, however our search did include any studies with incidental uptake in ectopic ACTH producing tumors on SSTR PET/CT [39]. One study by Parghane et al. reported four patients with SSTR uptake in skull base paragangliomas in a study population of NET patients referred to 177Lu-DOTATATE Peptide Receptor Radionuclide Therapy (PRRT), a study population we suspect has a higher prevalence of paraganglioma of unknown malignant potential. We therefore suspect our calculated risk of malignancy of cranial incidental findings to be higher than in a general population undergoing a SSTR PET-scan.

Incidental spine SSTR PET/CT findings were seldom malignant when excluding known NET metastases. It should be noted that almost all cases included in our study were from the study by Gauthe et al. and represent cases of vertebral hemangioma (93%). In this study the authors reported even more incidental findings in degenerative spinal lesions, but unfortunately these cases could not be included in our final synthesis as the DOTATOC-avidity and exact number of study subjects with benign non-vertebral hemangioma lesions were unclear.

Although rarely encountered, incidental breast SSTR PET/CT findings carried the highest risk of malignancy (67%). Based on our results, rapid diagnostic evaluation of incidental breast SSTR PET/CT is advised (Table 5). The study by Elgeti et al. is the only retrospective cohort study on incidental breast uptake on SSTR PET/CT with an estimated incidence of 6% which is higher than the reported incidental breast uptake by FDG PET of approximately 0.6% of all FDG PET/CT scans with a wide variety in reported malignancy risk ranging from 27–85% [40, 41].

**Table 5:** Overview pooled incidental malignant SSTR PET findings

	**Total malignant findings, n**	**Percentage of incidental findings (%)**
**Overall**	17	13%
**Location**	**Total number of malignant findings / total number of incidental findings in respective location**	**Percentage of incidental findings in respective organ, %)**
Breast	4 / 6	67%
Cranial	5 / 26	19%
Thyroid	5 / 65	8%
Spine	1 / 30	3%
Miscellaneous	2 / 7	29%

Although SSTR-tracers has shown lymphomas to be SSTR-tracer avid, our search revealed only one case of incidentally detected of non-Hodgkin lymphoma. Lymphomas should therefore also be considered a potential pitfall although it is rarely incidentally encountered according to our systematic review [42, 43].

Our study has several limitations. Most importantly, the present systematic review revealed a lack of high-quality data preventing the planned meta analyses, the vast majority were single center studies on specific organs and case reports. Similarly, a publication bias is present: there are relatively few studies reporting SSTR PET/CT and—on a case level—there are even fewer studies reporting incidental SSTR PET findings. As highlighted above, some incidental findings could not be included in our final synthesis due to lack of information in the reported cases. One must suspect that SSTR PET incidental findings are heavily underreported leading to publication bias in our study. Also, the majority of included studies are case report which will most likely skew the results of our review towards malignant or rare pathologies. Additionally, some organs are more well-studied than others. Most of the included study subjects were from retrospective cohort studies conducted at single centers focusing on a single organ. These studies, although valuable, can result in a biased study population when conducting a systematic review on incidental findings on SSTR PET in general.

Also, due to limited patient information in some cases, it is doubtful whether these reported incidental findings were true SSTR PET incidental findings or represented already known or secondary lesions to the primary NET initially indicated on the SSTR PET. Therefore, on a case level, our study carries a risk of selection bias. As described above, some cases from excluded studies could have impacted our study if they had met the pre-defined inclusion and exclusion criteria.

## Conclusion

We performed a systematic review of the available studies on incidental SSTR PET findings and found that most atypical findings of uptake on SSTR PET were located in the thyroid gland, spine, and cranially. The risk of malignancy was greatest for incidentalomas located in the breasts and in patients with focal uptake in the thyroid gland and in the brain. The present study can provide guidance in the interpretation of atypical findings on SSTR PET/CT and in the counseling of clinicians.

## Supplementary Information

**Additional file 1.**

## Data Availability

All data generated or analysed during this study are included in the present article. Search strings are provided in supplementary materials.

## Abbreviations

PET/CT: Positron emission tomography / computer tomography
SSTR: Somatostatin receptor
SSTR PET/CT: ^68^ Ga-DOTA-conjugated somatostatin receptor-targeting peptide PET/CT
NET: Neuroendocrine tumors
DOTA: 1,4,7,10-Tetraazacyclodecane-1,4,7,10-tetraacetic acid
PRRT: Peptide Receptor Radionuclide Therapy
