# Supplementary material for: Prevalence and significance of incidental findings on 68 Ga-DOTA-conjugated somatostatin receptor-targeting peptide PET/CT: a systematic review of the literature
Source: Cancer Imaging. 2022 Sep 3;22:44. doi: 10.1186/s40644-022-00484-0 (PMC9441055; doi:10.1186/s40644-022-00484-0)
Supplement: Supplementary file 1 — Additional file 1. [file 40644_2022_484_MOESM1_ESM.pdf]

Search Name:
Date Run: 02/06/2020 12:56:12
Comment:

ID Search Hits
#1 MeSH descriptor: [Tyrosine] explode all trees 2646
#2 (somatostatin):ti,ab,kw (Word variations have been searched) 2143
#3 MeSH descriptor: [Octreotide] explode all trees 689
#4 (lanreotide):ti,ab,kw (Word variations have been searched) 323
#5 (pentetreotide):ti,ab,kw (Word variations have been searched) 16
#6 (edotreotide):ti,ab,kw (Word variations have been searched) 7
#7 (DOTA*):ti,ab,kw (Word variations have been searched) 278
#8 #1 OR #2 OR #3 OR #4 OR #5 OR #6 OR #7 5419
#9 MeSH descriptor: [Incidental Findings] explode all trees 25
#10 (incidentalom*):ti,ab,kw (Word variations have been searched) 29
#11 (((Incidental OR unexpected) AND (finding* OR result* OR discover* OR detect* OR diagnos*))):ti,ab,kw (Word variations have been searched) 5779
#12 #9 OR #10 OR #11 5802
#13 MeSH descriptor: [Positron-Emission Tomography] explode all trees 959
#14 MeSH descriptor: [Tomography, Emission-Computed, Single-Photon] explode all trees 1001
#15 (PET):ti,ab,kw (Word variations have been searched) 6561
#16 MeSH descriptor: [Tomography, X-Ray Computed] explode all trees 4880
#17 ((positron emission tomograph*)):ti,ab,kw (Word variations have been searched) 4059
#18 ((single photon emission tomograph*)):ti,ab,kw (Word variations have been searched) 1919
#19 ((X-ray computed tomograph*)):ti,ab,kw (Word variations have been searched) 5958
#20 #13 OR #14 =R #15 OR 16 OR 17 OR #18 OR #19 345445
#21 #8 AND #12 AND #20 12

Embase®

Embase Session Results

No.	Query	Results
**#34**	**#18** AND **#24** AND **#33**	171
**#33**	**#25** OR **#26** OR **#27** OR **#28** OR **#29** OR **#30** OR **#31** OR **#32**	359,280
**#32**	(**'x ray'** NEAR/5 **computed** NEAR/5 **tomograph***):ti,ab,kw	13,442
**#31**	(**single** NEAR/5 **photon** NEAR/5 **emission** NEAR/5 **tomograph***):ti,ab,kw	23,710
**#30**	(**positron** NEAR/5 **emission** NEAR/5 **tomograph***):ti,ab,kw	81,459
**#29**	**'positron emission tomography-computed tomography'**/exp	29,048
**#28**	**'single photon emission computed tomography'**/exp	68,802
**#27**	**pet**:ti,ab,kw	174,965
**#26**	**'positron emission tomography'**/exp	163,709
**#25**	**'x-ray computed tomography'**/exp	54,141
**#24**	**#19** OR **#20** OR **#21** OR **#22** OR **#23**	52,457
**#23**	((**incidental** OR **unexpected**) NEAR/5 (**finding*** OR **result*** OR **discover*** OR **detect*** OR **diagnos***)):ti,ab,kw	40,927
**#22**	**incidentalom***:ti,ab,kw	3,360
**#21**	**'adrenal incidentaloma'**/exp	1,247
**#20**	**'incidentaloma'**/exp	2,402
**#19**	**'incidental finding'**/exp	17,233
**#18**	**#1** OR **#2** OR **#3** OR **#4** OR **#5** OR **#6** OR **#7** OR **#8** OR **#9** OR **#10** OR **#11** OR **#12** OR **#13** OR **#14** OR **#15** OR **#16** OR **#17**	315,171
**#17**	**dota***:ti,ab,kw	10,780
**#16**	**edotreotide**:ti,ab,kw	19
**#15**	**pentetreotide**:ti,ab,kw	743
**#14**	**lanreotide**:ti,ab,kw	1,595
**#13**	**somatostatin**:ti,ab,kw	38,441
**#12**	**tyrosine**:ti,ab,kw	222,429
**#11**	**'edotreotide ga 68'**/exp	208
**#10**	**'pentetreotide'**/exp	1,661
**#9**	**'pentetreotide in 111'**/exp	2,128
**#8**	**'lantreotide'**/exp	11
**#7**	**'indium 111 octreotide'**/exp	29
**#6**	**'octreotide'**/exp	22,013
**#5**	**'somatostatin receptor'**/exp	9,187
**#4**	**'somatostatin'**/exp	29,255
**#3**	**'tyrosine'**/exp	56,568
**#2**	**'gallium dotatoc ga 68'**/exp	23
**#1**	**'gallium dotatate ga 68'**/exp	590

ELSEVIER

© 2020 RELX Intellectual Properties SA. All rights reserved.
Embase, RELX Group and the RE symbol are trade marks of RELX Intellectual Properties SA, used under license.

History and Search Details

Search	Actions	Details	Query	Results	Time
#6			Search: **((("gallium Ga 68 dotatate" [Supplementary Concept] OR "Tyrosine"[Mesh] OR Somatostatin[TW] OR "Octreotide" [Mesh] OR Octreotide[TW] OR "indium 111-DOTA-lanreotide" [Supplementary Concept] OR DOTA*[TW] OR "lanreotide" [Supplementary Concept] OR lanreotide[TW] OR lanreotide[TW] OR pentetreotide[TW] OR edotreotide[TW] OR "Edotreotide" [Supplementary Concept] OR "Ga(III)-DOTATOC" [Supplementary Concept] OR "Receptors, Somatostatin"[Mesh])) AND (("Incidental Findings"[Mesh] OR "Adrenal incidentaloma" [Supplementary Concept] OR incidentalom*[TW] OR ((incidental[TW] OR unexpected[TW]) AND (finding*[TW] OR result[TW] OR results[TW] OR discover*[TW] OR detect*[TW] OR diagnos*[TW]))))) AND (("Positron-Emission Tomography"[Mesh] OR PET[TW] OR (positron-emission tomograph*[TW]) OR "Tomography, X-Ray Computed"[Mesh] OR "Tomography, Emission-Computed, Single-Photon"[Mesh]))**	105	06:11:43
#5			Search: **("Positron-Emission Tomography"[Mesh] OR PET[TW] OR (positron-emission tomograph*[TW]) OR "Tomography, X-Ray Computed"[Mesh] OR "Tomography, Emission-Computed, Single-Photon"[Mesh])**	549,089	06:10:38
#2			Search: **("Incidental Findings"[Mesh] OR "Adrenal incidentaloma" [Supplementary Concept] OR incidentalom*[TW] OR ((incidental[TW] OR unexpected[TW]) AND (finding*[TW] OR result[TW] OR results[TW] OR discover*[TW] OR detect*[TW] OR diagnos*[TW])))**	80,667	04:23:56
#1			Search: **("gallium Ga 68 dotatate" [Supplementary Concept] OR "Tyrosine"[Mesh] OR Somatostatin[TW] OR "Octreotide" [Mesh] OR Octreotide[TW] OR "indium 111-DOTA-lanreotide" [Supplementary Concept] OR DOTA*[TW] OR "lanreotide" [Supplementary Concept] OR lanreotide[TW] OR lanreotide[TW] OR pentetreotide[TW] OR edotreotide[TW] OR "Edotreotide" [Supplementary Concept] OR "Ga(III)-DOTATOC" [Supplementary Concept] OR "Receptors, Somatostatin"[Mesh])**	130,751	04:20:12

Web of Science

Clarivate Analytics

Tools Searches and alerts Search History Marked List

Select a database All Databases

Basic Search Cited Reference Search **Advanced Search**

Use field tags, Boolean operators, parentheses, and query sets to create your query. Results will appear in the Search History table at the bottom of the page.(Learn more about Advanced Search)

Example: TS=(nanotub* AND carbon) NOT AU=Smalley RE
#1 NOT #2 more examples | view the tutorial

Search

Booleans: AND, OR, NOT, SAME, NEAR

Field Tags:

**TS**= Topic	**SO**= Publication Name [Index]
**TI**= Title	**DO**= DOI
**AU**= Author [Index]	**PY**= Year Published
**AI**= Author Identifiers	**AD**= Address
**GP**= Group Author [Index]	**SU**= Research Area
**ED**= Editor	**IS**= ISSN/ISBN

Timespan

All years (1900 - 2020)

More settings

Select Databases

- Web of Science Core Collection
- KCI-Korean Journal Database
- MEDLINE®
- Russian Science Citation Index
- SciELO Citation Index

Auto-suggest publication names

On

Search language to use

Auto select

(To save these permanently, sign in or register.)

Search History:

Set Results Save History / Create Alert Open Saved History

Combin
AND
Comb

Set	Results	
# 4	107	#3 AND #2 AND #1<br>*Databases= WOS, KJD, MEDLINE, RSCI, SCIELO Timespan=All years*<br>*Search language=Auto*
# 3	849,063	TS=(PET OR (single-photon tomograph*) OR (positron-emission tomograph*) OR (X-ray computed tomograph*))<br>*Databases= WOS, KJD, MEDLINE, RSCI, SCIELO Timespan=All years*<br>*Search language=Auto*
# 2	137,874	TS=(((incidental OR unexpected) (finding* OR result* OR discover* OR detect* OR diagnos*)) OR incidentalom*)<br>*Databases= WOS, KJD, MEDLINE, RSCI, SCIELO Timespan=All years*<br>*Search language=Auto*
# 1	64,776	TS=(tyrosin OR somatostatin OR lanreotide OR pentetreotide OR edotreotide OR DOTA*)<br>*Databases= WOS, KJD, MEDLINE, RSCI, SCIELO Timespan=All years*<br>*Search language=Auto*

AND
Comb
